# Mo-Based Layered Nanostructures for the Electrochemical Sensing of Biomolecules

**DOI:** 10.3390/s20185404

**Published:** 2020-09-21

**Authors:** Rayhane Zribi, Giovanni Neri

**Affiliations:** Department of Engineering, University of Messina, C.da Di Dio, I-98166 Messina, Italy; zribi.rayhane@gmail.com

**Keywords:** Mo-layered compounds, nanosheets, electrochemical sensors, biomolecules

## Abstract

Mo-based layered nanostructures are two-dimensional (2D) nanomaterials with outstanding characteristics and very promising electrochemical properties. These materials comprise nanosheets of molybdenum (Mo) oxides (MoO_2_ and MoO_3_), dichalcogenides (MoS_2_, MoSe_2_, MoTe_2_), and carbides (MoC_2_), which find application in electrochemical devices for energy storage and generation. In this feature paper, we present the most relevant characteristics of such Mo-based layered compounds and their use as electrode materials in electrochemical sensors. In particular, the aspects related to synthesis methods, structural and electronic characteristics, and the relevant electrochemical properties, together with applications in the specific field of electrochemical biomolecule sensing, are reviewed. The main features, along with the current status, trends, and potentialities for biomedical sensing applications, are described, highlighting the peculiar properties of Mo-based 2D-nanomaterials in this field.

## 1. Introduction

Recently, nanotechnologies have enabled the manipulation of materials on a nanoscale, giving birth to new products with novel characteristics and properties. Two-dimensional (2D) nanomaterials, comprising individual nanosheets or layered multi-sheet planar materials, have been among the most intriguing nanomaterials of the last ten years. These thin 2D nanomaterials have special characteristics and properties that make them different from bulk ones [1,2]. One of the most attractive properties of this kind of material is linked to the number of layers or sheets, which can influence in a remarkable way almost all their properties, especially the electrical ones. The second important aspect is their large area to thickness ratio, with the consequence that the surface area is very high. Lastly, 2D nanomaterials have single or multiple layers with strong in-plane and weak out-plane bonding as atomically ordered networks. The combination of these microstructural characteristics confers on them, among other things, large adsorption capacity and strong surface reactivity, causing a big enhancement in their electrical, catalytic, and electrocatalytic properties with respect to bulk materials. 

Among the 2D nanomaterials, graphene, the first discovered 2D carbon material coming from the exfoliation of graphite, is attracting significant interest; but its major disadvantage is the lack of bandgap, which limits its use in some applications. In contrast to graphene, several 2D inorganic nanomaterials possess a sizable band gap and high reactivity towards gaseous species, including under ambient conditions, promising interesting applications in correlated fields [3]. The two-dimensional electron confinement of ultra-thin 2D nanomaterials, also leads to very interesting electrical properties compared to other nanostructures with different morphologies. All of these significant properties make the use of 2D materials the best choice in various fields such as sensing, energy-storage, drug carriers, diseases treatment, diagnosis, and therapy [4,5,6,7,8], which are expected to cause an enormous impact on human life within the near future. Impressive technical breakthroughs could thus be achieved for manufacturing state-of-the-art devices, with outstanding performance improvements from an engineering point of view.

The sensing of biomolecules by means of electrochemical sensors is one of the utmost research fields today, due to the strong driving force coming from biomedical and health care market sectors [9]. Detection of relevant biomolecules (among others, H_2_O_2_, glucose, neurotransmitters, vitamins, etc.) in body fluids (blood, urine, saliva, sweat) with these devices represents a simple way for acquiring information on health problems, helping in the prevention, control, and therapy of many diseases. The sensing mechanism of electrochemical sensors is based on the electrocatalytically activated redox processes of oxidation/reduction involving the target biomolecule (analyte) on the surface of a working electrode (WE). On the WE is present a receptor element, specifically recognizing the analyte. The molecular recognition reaction between the analyte and the receptor element changes the electrochemical properties of the sensor interface, thereby used as the sensor response. To collect the current originating from the redox events in the proper way, the electrochemical sensors are typically arranged in a two (WE and a counter electrode, CE) or three electrode configuration, with the additional presence of a reference electrode (RE). The WE assumes the key role in catalyzing the electrocatalytic processes. A number of different materials (enzyme, metal oxides, metal nanoparticles, etc.) can be used to improve the performance of the bare electrode, usually manufactured in carbon or gold. Nowadays, thanks to a large variety of nanomaterials that possess extremely enhanced structural (e.g., surface area, porosity) and electrical properties (e.g., large conductivity), the mass and charge transfers of the modified electrode are markedly maximized. Thin sheet-like 2D materials, exhibiting larger lateral size (>100 nm to few μm) with a few atoms of thickness (<5 nm), are highly desired for the development of efficient electrochemical sensors. Therefore, the high sensing performance necessary for entering the market, in order to replace commercial lab instruments devoted to biomolecule detection, can be reached more easily with respect to bulk electrode materials [10]. A number of nanomaterials with different morphologies and sizes have been proposed to be integrated into electrochemical sensing platforms for point-of-care solutions [11,12]. 

Layered inorganic nanomaterials, because of their unique electrical and electrocatalytic activity, have also been extensively examined for this purpose [13,14]. The developed devices show great potential, because their sensing characteristics can be modulated by the proper use of layered nanomaterials based on different inorganic elements [15,16,17]. In fact, the presence of inorganic elements with multiple oxidation states favors the redox reactions, which are at the basis of the functioning of electrochemical sensors. In addition, the precise control of the structure, dimension, and number of layers of 2D material-based electrode is an efficient way to improve their electrochemical performance. Indeed, the sensing characteristics of 2D materials are also usually layer-dependent and this factor introduces a new variable, which can be exploited to tailor the sensing device for a specific use [18,19]. 

**Figure 1:**
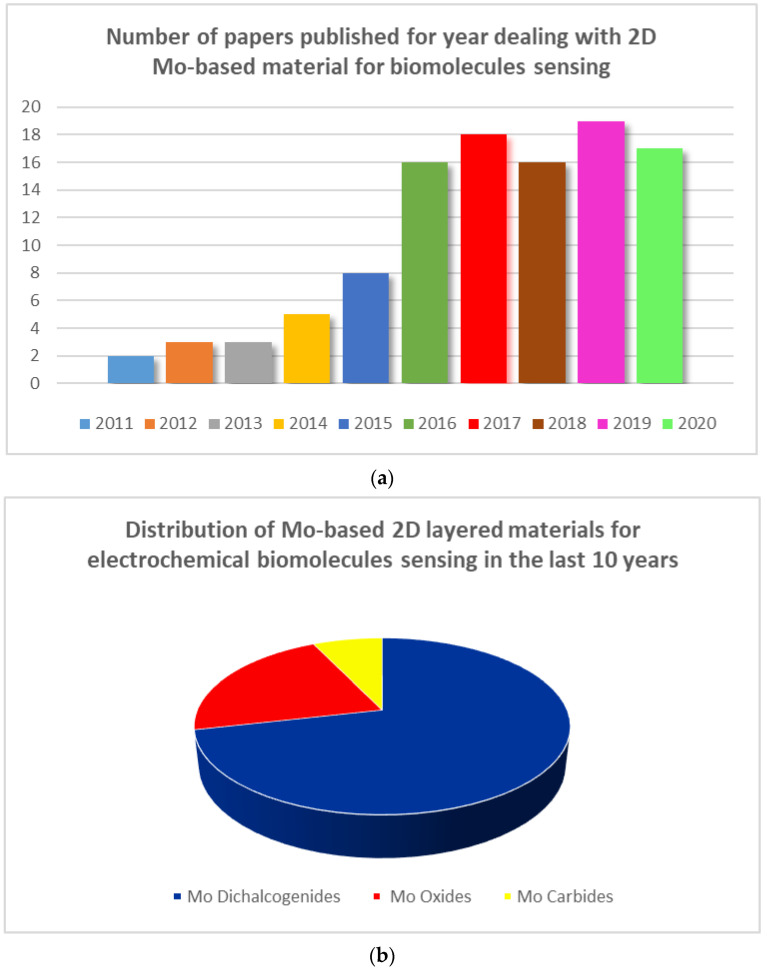
(**a**) Number of papers mentioning the use of molybdenum (Mo)-based materials for biomolecule sensing; (**b**) Distribution of Mo-based 2D layered materials for electrochemical biomolecule sensing.

The ability of molybdenum (Mo) to form many layered materials, is widely known, so molybdenum based (Mo-based) 2D layered materials have been exploited for developing electrochemical sensors [20,21]. Mo oxides, Mo dichalcogenides (e.g., MoS_2_), and carbides (i.e., MoC_2_) are the most investigated. However, despite the high importance, to our knowledge, no review dealing with Mo nanostructures for electrochemical sensing applications is found in the literature. A careful survey of the literature gives the opportunity to highlight their promising utilization in the field of electrochemical biomolecule sensing. The results of this investigation are summarized here. In Figure 1a is reported the noticeable growth in terms of number of papers published per year regarding 2D Mo-based materials employed for biomolecule sensing. Figure 1b highlights the distribution of different Mo nanostructures used; it is clearly noted that the Mo-dichalcogenides have been the most investigated.

Due to the strong interest, in this feature paper we review the various aspects of these layered materials ranging from synthesis methods, and structural and electronic characteristics to the relevant electrochemical properties in the specific field of electrochemical biomolecule sensing. The main features will be described, allowing a critical analysis of the current status, trends, and potentialities of Mo-based 2D-nanomaterials in biosensor applications.

## 2. Mo-Based 2D Layered Materials

Molybdenum can form a series of inorganic and organometallic compounds [22]. In these compounds, molybdenum exhibits multiple oxidation states, from −4 to +6, with this latter the most stable. These different oxidation states make molybdenum compounds very promising for many applications in electrochemistry. Most of these compounds, listed below, are in the form of 2D layered materials. 

### 2.1. Mo Oxides (MoO_3_ and MoO_2_)

In the last few years, molybdenum oxide (MoOx) nanomaterials have been proposed for many applications because of their physical, electrical, and chemical properties [23,24]. Different molybdenum oxides are among the most versatile electronic oxides [24] and also find applications in catalysis, sensors, biosystems, and so on. These compounds present various stoichiometries, ranging from full stoichiometric MoO_3_ as well as MoO_3−x_, where 2 < x < 3, and eventually semi-metallic MoO_2_. One of the main differences between these two types of MoOx is the bandgap, in which MoO_3_ has a wider bandgap [25]. Usually, MoO_3_ is found in three different crystalline structures: orthorhombic, monoclinic, and hexagonal phases, whereas MoO_2_ crystalizes in the monoclinic structure. MoO_3_ nanosheets have unique outer-d valence electrons which can be exchanged in electrochemical reactions. However, as the degree of reduction increases, the lattice may collapse and the layered structure is lost. 

### 2.2. Mo Dichalcogenides (MoS_2_, MoSe_2_, MoTe_2_)

Transition metal dichalcogenides (TMDCs) have become the center of research interest around the world in the last few years due to their important properties, such as high carrier mobility, high thermal conductivity and, unlike graphene, the transition from indirect bandgap (bulk) to direct bandgap (monolayer). TMDCs are inorganic layered compounds with strong interplane bonding and weak out-of-the-plane interactions. There are over 40 different types of transition metal dichalcogenides with MX_2_ as a stoichiometry, in which M is a transition metal and X is a chalcogen, such as sulfur (S), tellurium (Te), or selenium (Se). Regarding molybdenum dichalcogenides, MoS_2_ has been the most investigated and important member of this family, and is generally present in the hexagonal and rhombohedral phases. 1T-MoS_2_ is a new and metastable crystalline phase coming from modification of molybdenum disulfide. MoTe_2_ exists in three crystalline forms with rather similar layered structures: hexagonal α, monoclinic β, and orthorhombic β’. Concerning MoSe_2_, it is a trigonal prismatic metal that changes to octahedral after exfoliation. All Mo dichalcogenides change from indirect to direct band gap when they pass from 3D to 2D. 

### 2.3. Molybdenum Carbides (MoC, Mo_2_C)

After the discovery of Ti_3_C_2_ in 2011, 2D transition metal carbides, carbonitrides, and nitrides, known as MXenes, have been much investigated for their interesting characteristics. They are among the newest materials of the 2D family. MXenes are synthesized by selective etching of the “A” layers from layered ternary carbides [26]. Among these, Mo based MXenes such as molybdenum carbides (Mo_2_C and MoC) are the most promising. Molybdenum carbides were found in three structures: cubic δ-MoC, hexagonal α-MoC, and orthorhombic β-Mo_2_C [27,28]. Mo_2_C possesses interesting properties, such as good electrical conductivity, thermal stability, and elevated surface area [29].

### 2.4. Hybrid Structures

Notwithstanding the excellent properties displayed by the layered Mo compounds, they can present some drawbacks for the present application, such as poor intrinsic conductivity and the tendency for the sheets aggregation [30]. The functionalization of layered Mo compounds, with suitable organic and/or inorganic components, leading to the formation of hybrid nanocomposites, is one of the most effective approaches to overcome these limitations. Graphene (GN) is an excellent templating material to prepare Mo-based hybrid composite 2D nanostructures [31,32]. A series of other hybrid structure have also been synthesized, with the common characteristic of displaying enhanced properties with respect to single constituents [33,34,35].

Heterostructures based on 2D Mo materials can be synthesized by the layer-by-layer stacking method [34]. Recently, Yang and coll. proposed a facile method to synthesize a MoO_x_/Mo_2_C heterostructure, directly oxidizing Mo_2_C to MoO_x_ by an in situ technique [35]. The Mo_2_C deposited by chemical vapor deposition (CVD) shows high stability (below 350 °C in O_2_), demonstrating good thermal stability. 

## 3. Synthesis Methods of Mo-Based 2D Layered Materials

**Figure 2:**
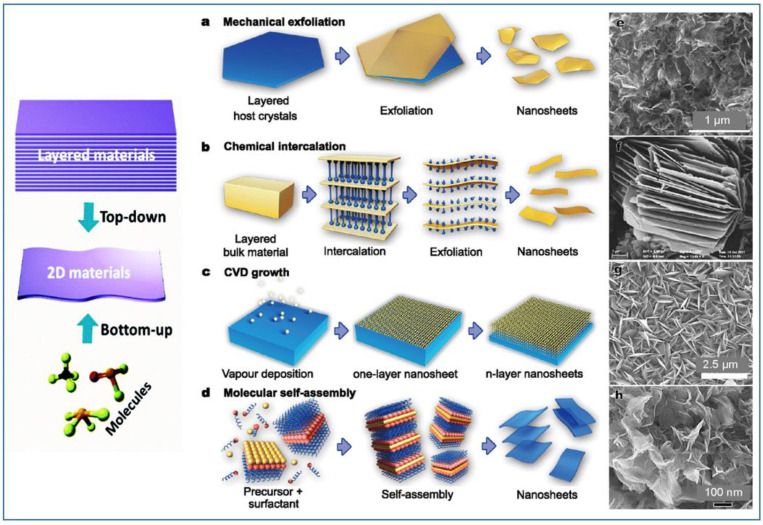
Examples of top-down and bottom-up strategies for the synthesis of 2D layered nanostructures: (**a**) mechanical exfoliation of layered host materials, (**b**) chemical intercalation induced exfoliation, (**c**) chemical vapor deposition (CVD) growth, and (**d**) wet-chemical self-assembly. Scanning electron microscopy (SEM) images of 2D nanomaterials obtained with the corresponding techniques: (**e**,**g**,**h**) TiO_2_ and (**f**) VO_2_ nanosheets.

The production of high-quality, scalable, and cost-effective layered nanomaterials is one of the prerequisites for developing reliable electrochemical sensors and exploiting their commercial applications. Usually, the methods for the preparation of layered or nanosheets can be divided into two types: “bottom-up” and “top-down”. For the “bottom-up” growth, both chemical and physical methods have been developed. The most recognized “top-down” method was the exfoliation technique, starting from three-dimensional layered materials in bulk. Figure 2 reports a composite picture showing some of these techniques for preparation, and examples of typical nanomaterials obtained [36]. 

A brief summary of these techniques of preparation, with special emphasis to 2D Mo-based materials, is reported below.

### 3.1. Atomic Layer Deposition (ALD)

Atomic layer deposition (ALD) is a well-known deposition technique for the deposition of thin films, based on the use of chemical processes occurring between reagents in a gas phase. The reactant precursors, reacting on the surface of a substrate in a sequential and self-limiting manner, lead to growth of a thin film. This method is also used for the synthesis of 2D nanosheet layered materials by controlling the number of depositions [37]. This technique has a lot of advantages, like high-quality films, conformality, and low temperature processing. On the other hand, it also presents some disadvantages, such as the long time required for the chemical reactions to occur for the growth of the material, and the waste of the material.

### 3.2. Pulsed Laser Deposition (PLD), Sputtering and Spray Pyrolysis

These techniques are relatively general and simple physical methods for the synthesis/deposition of thin films on different substrates. PLD uses a high-power laser to vaporize a target placed close to the substrate inside the vacuum chamber. This method was applied in the synthesis of several 2D Mo-based materials [38,39], because it is fast and, at the same time, less costly and highly reliable. Despite these advantages, layers of materials deposited by PLD have high defects and uneven coverage, not well suited for large-scale film growth. Radio frequency magnetron sputtering has been proposed for large scale deposition of homogeneous two dimensional MoS_2_ [40]. Spray pyrolysis has been also used for obtaining layered materials. The method is simpler and cheaper compared to others cited in this paragraph and works as following: a solution containing the reactive precursors is vaporized and then sprayed using a nebulizer on the substrate. The substrate is maintained at high temperature, allowing the activation of the reaction between the precursors on the substrate surface to form a film. The process can be performed in air and/or in a reaction chamber under vacuum. By this method the characteristics of the deposited film (particle size, shape and thickness, etc.) are obtained by controlling the sprayer parameters (energy, size of the droplet precursors, spray duration, distance between the spray and the substrate), and the furnace and substrate temperature [41,42]. 

### 3.3. Chemical Etching

This process is mostly used to obtain MXenes. For preparing one or few atomic layers of these compounds, layered ternary carbides and nitrides, called MAX, are used. By using hydrofluoric acid (HF), or other chemical etching solutions, the single A layers (mostly Al) are etched to obtain MXenes [26]. For example, Meshkian and coll. obtained several atomic layers etching two Ga layers from Mo_2_Ga_2_C in order to obtain Mo_2_C [43,44]. Chemical etching is a low cost, simple, and highly selective process but, at the same time, the repeatability is not guaranteed because of the strong influence of the temperature, the concentration of etchant, and problems deriving from chemical contamination.

### 3.4. Chemical Vapor Deposition (CVD)

Chemical vapor deposition (CVD) is a process by which a thin film is deposited by gas/vapor phase precursors. The reaction and/or decomposition of precursors occurs on the surface of the heated substrate, allowing the growth of a thin layer. Varying the experimental conditions, the nature of substrate, its temperature, the composition of the of reaction gas, the flow of gas, or its pressure, materials, different in composition and morphology, can be grown [45]; although it’s not suitable for large scale fabrication of 2D material and not suitable for mixtures of materials. This method is very useful thanks to the possibility to obtain high growth rates, deposit materials which are hard to evaporate, and grow epitaxial films. In addition to that, it presents a very good reproducibility. In spite of these interesting advantages, CVD apparatus is complex, requiring generally high temperature, and some use of toxic and corrosive gases. 

### 3.5. Hydrothermal Synthesis

Hydrothermal synthesis is one of the most commonly used methods for the preparation of nanomaterials due to its versatility. It is environmentally friendly, even if it needs a costly autoclave. Other concerns are related to safety issues and usually the samples obtained are polydispersed. Midya et al. proposed a hydrothermal chemical approach to obtain highly crystalline few layer template-free 2H-MoS_2_ nanosheets in solution [46]. Peng et al. prepared Mo-dichalcogenides (MoS_2_ and MoSe_2_) by an hydrothermal method at a mild temperature of 150 to 180 °C [47]. 

### 3.6. Exfoliation

This strategy has drawn great scientific interest and occupied the attention of researchers as a low cost, efficient method to produce 2D materials on a large scale. Below, the different strategies of exfoliation have been listed. 

The basics of mechanical exfoliation by adhesive tapes is to stick them on the two sides of the bulk and repeatedly stripe to get thin layers. By the ball milling method 2D layered material is obtained thanks to a combination of grinding with large and small balls. Sandpaper was another interesting tool for mechanical exfoliation.

The chemical exfoliation method is the most common strategy to fabricate nanosheet materials, exploiting the reaction of layered materials with strong oxidants or other active compounds. By reacting with the layered materials, active compounds form functional groups or insert the intercalated ions between layers, swelling the layered materials, and causing the enlargement between the layers. A ultrasonic treatment is generally performed for obtaining the 2D nanosheets.

Electrochemical exfoliation is generally used to obtain, applying a suitable voltage, graphene from graphite. Ionic species are driven to intercalate into graphite forming gaseous species that expand and exfoliate individual graphene sheets [48].

**Figure 3:**
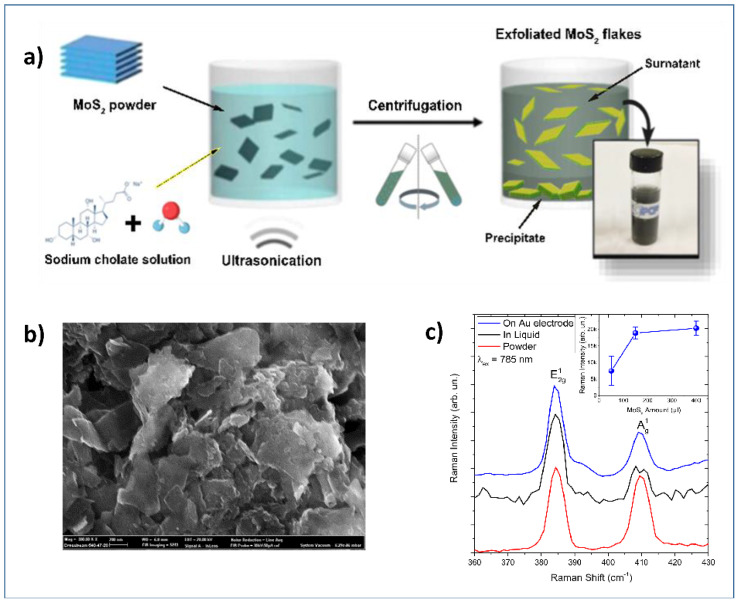
(**a**) Schematization of the ultrasonication process for obtaining 2D-MoS_2_ nanosheets; (**b**) SEM images showing 2D-MoS_2_ deposited on the surface of a gold electrode; (**c**) Raman spectra carried out at 785 nm. The Inset shows the intensity of the Raman band as a function of MoS_2_ loading. Adapted with permission from reference [49].

Ultrasonication of the bulk compounds in a liquid media has also been used for the synthesis of 2D materials. Two main factors can influence the exfoliation: the first one is the energy input to the bulk in liquid media by the bath sonication or probe sonication, and the second factor is the nature of the liquid media used [45,49]. Zribi et al. prepared 2D-MoS_2_ prepared via liquid ultrasonication (Figure 3a) for finalizing its use in electrochemical sensing [49,50]. A detailed characterization by SEM (Figure 3b) has been carried to confirm the preparation of nanosheets of MoS_2_ and their deposition on the gold electrode. 

Raman analysis (Figure 3c) was also carried out, confirming the 2D-dimensionality of the MoS_2_ deposited on the electrochemical platform, as demonstrated by spectra collected on the liquid dispersion (black lines), on powder (red lines), and on the MoS_2_ loaded gold electrode (blue line). The inset shows the intensity of the Raman band as a function of MoS_2_ loading. 

### 3.7. Miscellaneous Synthesis

**Figure 4:**
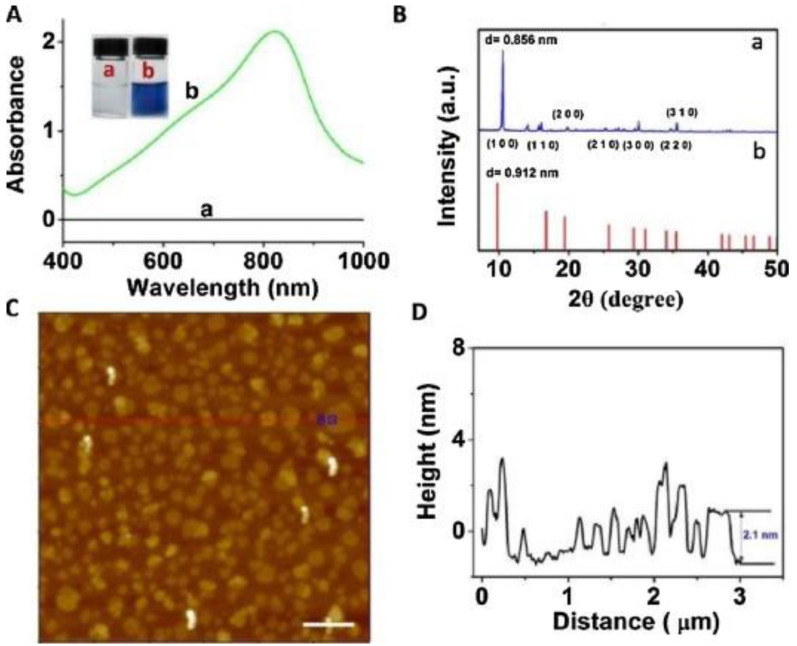
(**A**) UV–vis-NIR absorption spectra of (**a**) MoO_3_ nanosheet and (**b**) MoO_3−x_ nanosheet dispersions. (**B**) XRD pattern of (**a**) the MoO_3−x_ nanosheets, and (**b**) JCPDs card no. 21-0569 of hexagonal phase of MoO_3_. (**C**) AFM image of MoO_3−x_ nanosheets (scale bar, 500 nm). (**D**) Height profile. Reprinted with permission from reference [55].

In addition to the above mentioned synthesis methods for obtaining Mo-layered materials, more specific methods have been also reported. For example, these include the synthesis of MoO_3−x_ nanosheets by nonaqueous solvothermal method [51], supercritical carbon dioxide [52], and laser irradiation [53]. Song et al. obtained α-MoO_3_ by sintering, in a very efficient and environmentally friendly manner, aggregates of ammonium molybdate tetrahydrate (AMT) with polyethylene glycols (PEGs) [54]. Various α-MoO_3_ nanostructures can be obtained, suggesting that the surface structures and morphology can be controlled by changing various parameters. Among these, PEGs play a role in mediating the morphology of the obtained product, through the initial aggregation with AMT and the successive decomposition into α-MoO_3_ as the temperature rises. Li et al. reported the rapid and simple synthesis of MoO_3−x_ nanosheets, by an environmentally friendly method, by using ascorbic acid (AA) as a reducing agent under an acidic environment at room temperature [55]. Figure 4A,B show results obtained in the UV–vis-NIR characterization of (a) MoO_3_ nanosheets and (b) MoO_3−x_ nanosheet dispersions. AFM image of MoO_3−x_ nanosheets is also reported in Figure 4C, along with the height profile shown in Figure 4D.

## 4. Electrochemical Sensors

Electrochemical sensors represent the most versatile and highly developed devices for the sensing of biomolecules. Such devices hold a prominent position on the commercial stage, and have important applications in many fields. The main advantage of these sensors is the simplicity and the ease of use; these factors contributed to make electrochemical sensors the most used chemical sensors on the market. The sensing mechanism of electrochemical sensors is based on transduction of processes of oxidation/reduction of the target analyte which are electrocatalytically activated on the surface of bare- or modified-WE. Bare WE is generally in carbon or gold, but in practice it is preferably activated/modified with suitable activators/modifiers. In the enzymatic-type sensors, an enzyme is deposited on the WE. These sensors have the advantages of high sensitivity and selectivity towards the analytes to be detected. However, they generally have a high cost and short life-time due to instability of the enzyme. In order to overcome these main drawbacks, the working electrode is modified with suitable materials, which confer it enhanced electrocatalytic properties. In this specific case, nanomaterials possess the suitable characteristics necessary to improve the overall electrochemical processes of oxidation/reduction, increase the sensitivity, and minimize the drawbacks associated with selective detection and stability issues. In this respect, many Mo-based nanomaterials are able to provide interesting properties and their use promises exciting applications in this field [16]. 

**Figure 5:**
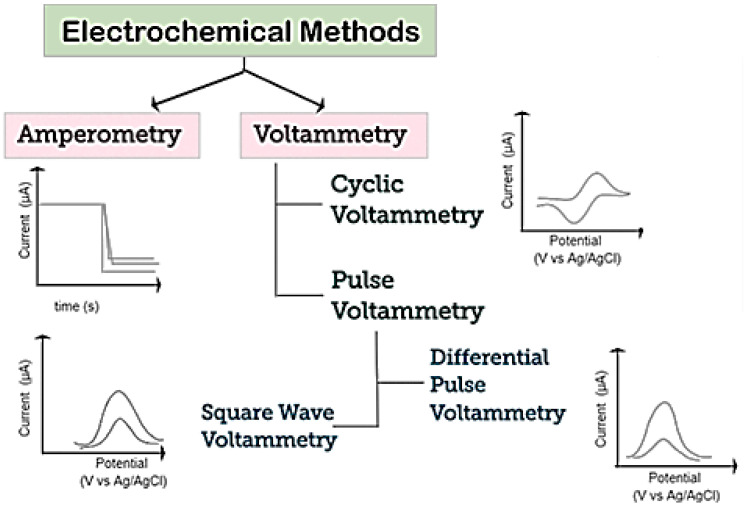
Schematization of electrochemical techniques used for detection of biomolecules. Adapted with permission from reference [56].

A schematization of various electrochemical techniques which can be used for the detection of biomolecules is reported in Figure 5. Voltammetry and amperometry are among the most common [56]. In both techniques, a constant, scanning, or pulsing potential is applied to the WE versus a reference electrode and the current is measured. 

Among voltammetric methods, cyclic voltammetry (CV), differential pulse voltammetry (DPV), and square-wave voltammetry (SWV) are the most popular. CV is generally used to study and investigate the electrochemical behavior of electroactive species. CV is performed by scanning, from an initial potential, at a fixed rate, to a switching potential at which the scan direction is reversed toward a final potential, and in measuring the resulting current. As a result a cycle CV (voltammogram) is registered by plotting the current vs. the potential applied.

Regarding amperometry, this technique uses a fixed potential and measures the variations in current over time, caused by electroactive species. The electrode potential is maintained at a constant level, sufficient to transfer electrons to/from the electroactive analytes. An electrical current is then developed, proportional to the concentration of analyte [57].

In addition to voltammetric methods mentioned above, electrochemical impedance spectroscopy (EIS), a technique mainly used today merely to characterize electrodes, can be also used for electroanalytical scopes. By this method, the electrical impedance of an analyte as a function of the frequency of an applied alternate electrical current, is measured. With this operating method, EIS can give an output that has been shown to regress linearly with analyte concentration.

In summary, the electrochemical devices utilized for the sensing of biomolecules are highly versatile and more competitive, compared to other sensor typologies such as optical, gravimetric, and so on. 

## 5. Mo-Based 2D Layered Materials for Electrochemical Biomolecules Sensing

In the last few years, electrochemical sensors have gained the interest of researchers for the detection of biomolecules, i.e., substances having a key role in the biochemical processes of living systems. From small molecular weight molecules, e.g., H_2_O_2_, to the larger, e.g., DNA, they can give direct or indirect evidence of some health problems (biomarkers), so their quantification in body fluids (blood, urine, saliva, sweat) is of utmost importance for the prevention, control, and therapy of many diseases. The next paragraphs list the most important biomolecules, along with the layered Mo-based electrochemical sensors investigated for their monitoring. 

### 5.1. Hydrogen Peroxide

Hydrogen peroxide is an important biomolecule circulating in our body, because it is a by-product of numerous enzymatic reactions occurring in living organisms [58]. Hydrogen peroxide is also produced in response to a number of cellular perturbations and/or stresses, thus, it is closely bound up with human health [59]. Indeed, when H_2_O_2_ is accumulated in cells at high concentrations, it leads to several damages, causing aging and various diseases. 

**Figure 6:**
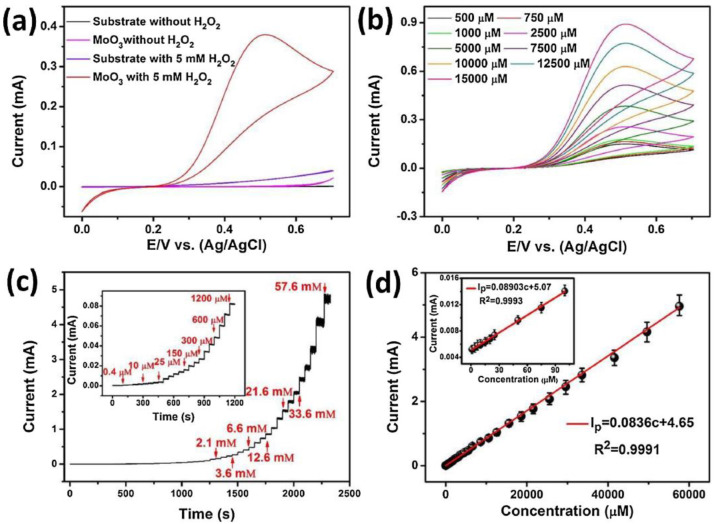
(**a**) Cyclic voltammetry (CV) of the bare substrate and atomic layer deposition (ALD)-developed 2D α-MoO_3_ in the absence and presence of 5 mM H_2_O_2_. (**b**) CV curves of 2D α-MoO_3_ at different concentration of H_2_O_2_. (**c**) Amperometric current response to H_2_O_2_ concentration variation. (**d**) Current vs. H_2_O_2_ concentration. Reprinted with permission from reference [64].

Various Mo-based 2D-nanomaterials have been reported for hydrogen peroxide detection by means of electrochemical methods [60,61,62,63,64]. Wei et al. synthesized 2D α-MoO_3_ nanostructures by an ALD technique [64]. The synthesized layers were composed of flat orthorhombic phase (α-MoO_3_) of about 35 nm. CV, EIS and chronoamperometry were performed to evaluate the electrochemical behaviors of 2D α-MoO_3_ sensors for H_2_O_2_ detection. Figure 6a,b reports data obtained in the presence and absence of H_2_O_2_ in 0.1 M PBS (pH = 7.0) at a scan rate of 10 mV/s. The data showed that H_2_O_2_ is easily electrocatalytically oxidized on the 2D α-MoO_3_ modified electrode. Exploiting this, the authors used amperometric analysis (Figure 6c,d), the simpler electrochemical method and the most suitable for market upgrading, for the quantitative determination of H_2_O_2_.

The good sensing performance, in terms of elevated sensitivity and selectivity, stability in the long-term, fast response/recovery time, and wide linear range with a low LOD, suggests the great potential of this sensor for practical utilization in highly sensitive H_2_O_2_ devices.

A microfiber hybrid structure of MoS_2_ nanosheets and graphite which display peroxidase-mimicking activity has been proposed for the quantification of hydrogen peroxide. Unlike the above 2D α-MoO_3_ nanostructures, on MoS_2_ nanosheets (compare also reference [60]) the sensing pathway relies on the electrocatalytic reduction of hydrogen peroxide. This fact highlights the different electrochemical properties displayed by the various 2D-Mo compounds, which is of valuable interest in diversifying the desired electrochemical applications. The biosensor has noticeable sensing performances, due to the synergistic enhancement of the synthetic nanozymes (few-layered MoS_2_ nanosheets) and immobilized natural peroxidase [65]. Li et al. developed a novel amperometric sensor for H_2_O_2_, using a layered hierarchical porous α-MoO_3_ and GO modified glass carbon electrode [66]. The synthesized α-MoO_3_ showed a wedge-shaped morphology, accumulated by micro-slices comprised of nanosheets and small particles, with an average size of 25 nm. 

### 5.2. Glucose

Glucose represents the primary source of energy for the body, in fact it is used by all cells, especially brain cells. Glucose is an important biomarker to be detected in human blood in order to help diabetics to maintain stable their level of glucose, so its detection is of utmost importance in health care [60]. In this context, Azharudeen et al. proposed a α-MoO_3_ nanoflake sensor for glucose monitoring [67]. A glucose sensor was fabricated using MoO_3_ nanoflakes modified with polyvinylpyrrolidone (PVP) as a capping molecule. The sensor operated in alkaline conditions, pH = 12. The MoO_3_/(6%)PVP sensor exhibited good selectivity, a LOD of 0.022 µM, and sensitivity of 86.42 µA mM^−1^ cm^−2^, which was related to the mesoporous nature of the nanocomposite. 

**Figure 7:**
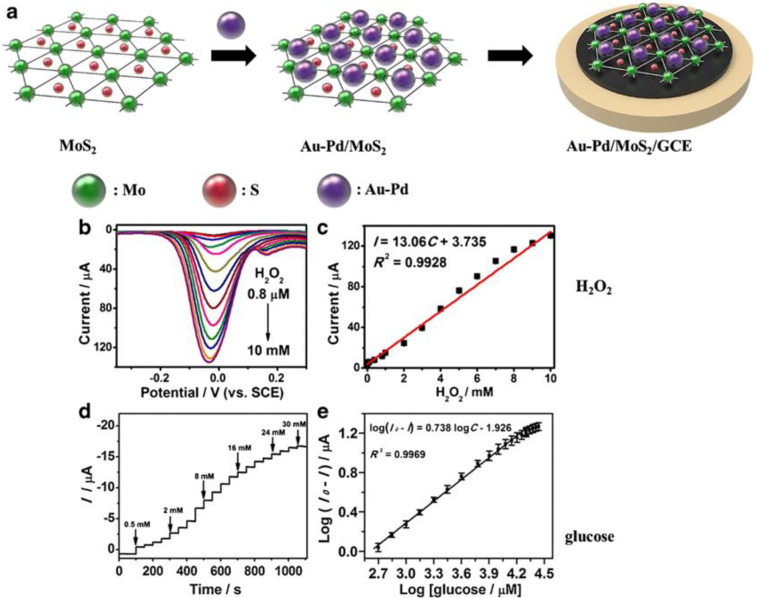
(**a**) Schematic representations of Au-Pd/MoS_2_/GCE sensor. (**b**) Differential pulse voltammetry (DPV) curves for different H_2_O_2_ concentrations. (**c**) DPV peak current as a function of H_2_O_2_ concentration. (**d**) Amperometric responses for different glucose concentrations. (**e**) Plot of amperometric response vs. the logarithm of glucose concentration. Reprinted with permission from reference [60].

MoS_2_-based electrochemical glucose biosensors have also been reported. Zhai et al. developed an enzyme-free sensing platform for monitoring glucose, by using a MoS_2_ micro-flower (through a hydrothermal method assisted by a surfactant, such as CTAB) modified glassy carbon electrode (GCE) [68]. A schematic representation of the Au-Pd/MoS_2_/GCE sensor developed by Li et al. for detecting hydrogen peroxide and glucose is shown in Figure 7 [60].

The glucose sensing properties of Mo layered materials can be further enhanced by combining them with metal NPs such as Pt, Au, Ni, or Cu, exploiting the high electrochemical oxidation to glucose of these additives [69,70]. Noble metals display large catalytic activity at physiological pH (near to 7), but they have a high cost compared to Cu or Ni. These latter, however, operate at higher pH (>9–10). For example, it is well known that the Ni^3+^/Ni^2+^ redox couple offers an exceptional catalytic activity in alkaline conditions [71]. Many studies have therefore been reported on the use of MoS_2_ nanosheets as a large area support to immobilize metal nanoparticles [69,72,73]. Huang et al. tested MoS_2_ nanosheets decorated with Cu. Combining the strong glucose oxidation of Cu and the large surface area and active edge sites of MoS_2_ nanosheets, the Cu/MoS_2_ nanocomposite showed high electrocatalytic activity towards glucose oxidation [74]. The sensitivity of 1055 μA mM^−2^ cm^−2^ of this glucose sensor was nearly double compared to the MoS_2_ micro-flower electrode. Furthermore, the detection range was linear of up to 4 mM, and selectivity against numerous interferent analytes was good. 

### 5.3. Neurotransmitters

**Figure 8:**
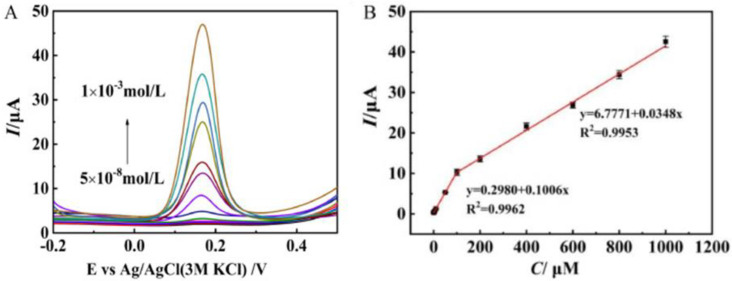
(**A**) DPV curves of S-MoSe_2_/NSG/Au/MIP/GCE to dopamine (DA) concentrations from 5 × 10^−8^ to 1 × 10^−3^ mol/L. (**B**) Peak current vs. DA concentration. Reprinted with permission from reference [83].

Neurotransmitters, also known as neuromediators, are biomolecules that ensure the transmission of messages from one neuron to another. Dopamine (DA) is one of them, regulating the reward and pleasure centers in human body. DA is also crucial for memory processes and motor skills, and is known to be implied in stress-related health problems, similarly to cortisol [75]. Various layered Mo materials-modified electrodes have been proposed for this purpose [76,77,78,79,80,81,82]. Zang et al. [83] synthesized, via a novel hydrothermal route, a few layer S-MoSe_2_/NSG with many rich-edge sites. This work demonstrated that the S-MoSe_2_/NSG/Au/MIPs modified GCE presents good sensitivity to DA (see Figure 8). The limit of detection (LOD) computed was 0.02 μM, with two linear ranges in the interval from 0.05 to 1000 μM. The sensors was successfully tested in real blood samples.

Pramoda and coworkers deposited MoS_2_-RGO composites, prepared by hydrothermal synthesis assisted by ultrasonication, on GCEs for selectively detecting DA and uric acid (UA) in the presence of ascorbic acid (AA) as the main interferent substance [84]. The enhanced performance of the MoS_2_-RGO/GCE sensor towards dopamine was related to the rise in surface area (up to 147 m^2^/g) and the facilitated electron transfer through the interaction of π-π with the graphene binding surface of DA.

### 5.4. Vitamins

Vitamins are considered to be vital constituents of our food supplements as their deficiency causes many detrimental health complications. They cannot be synthesized by the human body, and small quantities are required in the diet [85]. Among them, the most important involved in human health are the water soluble vitamins vitamin B2 or riboflavin (RB), vitamin C or ascorbic acid, and vitamin B9 or folic acid (FA). 

Hussain et al. [86] proposed a Mo_2_C-FA/GCE for the monitoring of FA. For the fabrication of the sensor, Mo_2_C was first synthesized by a facile chemical reduction method and deposited on the GCE. Then, FA was imprinted in the presence of pyrrole over GCE by electropolymerization. The proposed modified electrode showed good behavior, in terms of a wide detection range of FA, going from 0.01 μM to 120 μM, a low detection limit of about 4nM, good selectivity towards FA, with co-existing species in real samples, and also good repeatability (RSD; 1.6%) and stability. 

Mani et al. synthesized a GNS-MoS_2_-AuNPs nanocomposite and developed an electrochemical sensing method for the determination of folic acid [87]. They investigated in detail the morphological and electrochemical characteristics of the ternary nanocomposite. The ternary composite demonstrated good electrocatalytic ability (wide linear range of 50 nM–1150 μM, low detection limit of 38.5 nM) for folic acid, which has been attributed to the synergic effect between GNS, MoS_2_, and AuNPs. 

The practical applicability of the Mo-based 2D-nanomaterial electrodes has also been verified by conducting tests in human urine, as well as in human serum samples [87,88].

### 5.5. DNA

**Figure 9:**
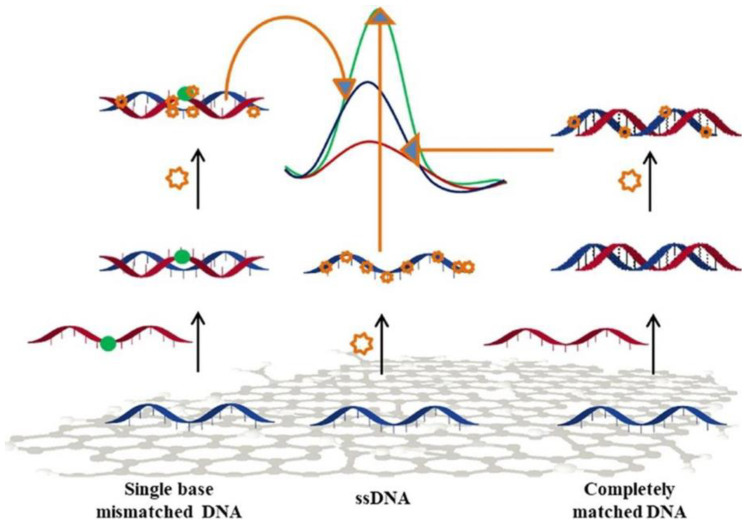
Scheme of the DPV signal of MoS_2_-polyaniline sensors with completely matched and single base mismatched DNA. Reprinted with permission from reference [90].

The detection and analysis of specific DNA sequences is of utmost importance in molecular diagnosis. Electrochemistry-based sensors offer suitable performance for the detection of selected DNA sequences. These sensors take advantage of interactions at nanoscale between the DNA target and the recognition layer at the electrode surface, through several electrochemical approaches [89]. Mo-based 2D materials have been used in DNA detection [90,91,92]. Dutta et al. [90] proposed a MoS_2_-polyaniline nanocomposite for developing a label-free sensor using an immobilized single stranded DNA. In this context, a few layers of MoS_2_ were first synthesized via the hydrothermal route. The sensor showed an important response, without labeling or any use of amplifiers, even at low concentration, like 10^−15^ M of target DNA, and had a wide linear range going from 10^−6^ to 10^−15^ M. The sensor also presented a good selectivity versus interferent analytes in serum samples, and recognized any positional mismatch in the targeted DNA (Figure 9).

Tian et al. [92] proposed a N-carboxymethyl chitosan/Mo_2_C nanocomposite for fabricating a sensor with large conductivity. This biosensor exhibited a detection range of 1.0 fM to 1.0 nM with a detection limit of 0.34 fM. The sensor performance, like good selectivity, reproducibility, stability, and a good response in human serum offer great potential for the early clinical diagnosis of various genetic diseases. 

### 5.6. Other Biomolecules

**Figure 10:**
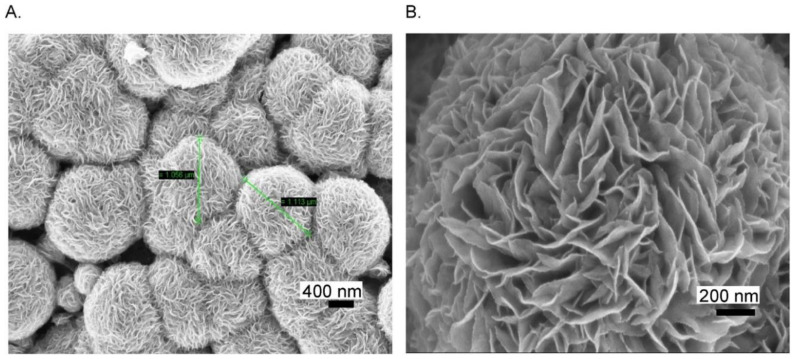
(**A**) Low and (**B**) high magnification FESEM images of MoS_2_ grown Al foil. Reprinted with permission from reference [93].

2D Mo-based sensors have also been reported for sensing biomolecules other than the ones previously mentioned. They could find a large use in the field of health care, owing to the importance of biomolecule detection. Citing an example, uric acid is a well-known byproduct present in our body due to the decomposition of purines. A high level of UA causes gout disease, kidney stones, or kidney failure. A flexible and a low-cost sensor based on MoS_2_ has been proposed by Sha et al. [93] for the determination of UA. First, MoS_2_ was synthesized by hydrothermal growth on aluminum foil. FESEM images taken at low and high magnification, Figure 10A,B, respectively, confirmed the MoS_2_ micro-flower like structure. 

The proposed sensor presents a wide dynamic range from 10 to 400 µM, a good sensitivity of about 98.3 × 10^−3^ A M^−1^, a low detection limit of 1.169 µM, a response time of <3 s, and excellent reproducibility. The efficiency of the sensor was exploited in the detection of uric acid in urine with good performance.

Functionalizing 2D MoS_2_ with Al, Cu, Sn, and Ti, Selvam et al. developed a low-cost, ultra-selective biosensor for the detection of four vital neurological drugs, aspirin, nicotine, caffeine, and tramadol, in saliva [94]. The different sensors were arranged in an array for fabricating an electronic tongue (E-Tongue). By pattern-recognition analysis of data coming from this sensor array, it could efficiently differentiate, in real-time, the drugs from one another in human saliva samples. 

Liu et al. used AuNPs/MoS2/AuNPs as a transducer for developing an ultrasensitive immune-sensor for cortisol, a biomarker of psychological or physical stress, detection at the point of care (POC). The system was portable, with the miniaturized differential pulse voltammetry (DPV) technique based on a smartphone, allowing the quantifying of salivary cortisol level during one day [95].

Mo-based 2D materials were also used in biosensors for monitoring: the growth factors BB, which regulate cell growth and division [96]; the toxicity of some inorganic species like nitrite ion, NO_2_^−^ [97,98]; rifampicin, an antibiotic useful in the treatment of a number of infections [99]; and some important factors for energy metabolism in the human body, like NADH [100]. Over and above these, Mo-based sensors were used for detecting germ cell tumors, mainly teratomas [101], lung cancer biomarkers like miRNA-182 [102], SCCA [103], and CEA [104], antimicrobial metronidazole [105], and other drugs [106,107,108,109,110,111,112,113]. 

## 6. Current and Future Perspectives

The above reported view on layered Mo-based sensors for biomolecule sensing has demonstrated the intensive exploration of these materials in the last years, which could lead to a number of promising applications in this field in the near future. Several Mo compounds contribute in this aspect because, in addition to the general properties of 2D materials, they display unique chemical and physical properties. MoS_2_ is undoubtedly the most researched 2D-Mo compound, but Mo-oxides and Mo-carbides have also gained much attention in this field. To make simpler the comparison among the varieties of Mo-based layered sensors currently used for biomolecule sensing, Table 1, Table 2 and Table 3 summarize their main characteristics.

A careful analysis of the data reported in Table 1, give us the opportunity to make some interesting observations. MoS_2_ is the preferred and most applied TMDC for these sensors, but wide applications in this field have been also demonstrated for MoSe_2_. Except in a few cases [49,61,75,93], Mo-based sensors rely on composite materials where the Mo-dichalcogenides are activated mainly with noble metals [60,83,84,91,97,102,114,115,116] and carbon nanostructures [77,78,84,96,97], like graphene or graphene oxide. In particular, AuNPs was the most considered noble metal additive, because of the high affinity of gold for the sulfur atom and the other chalcogenide elements, which allows the controlled synthesis of AuNPs on TMDC nanosheets. Gold nanoparticles are well-known efficient labels in electrochemical sensors, and act as viable materials to modify the surface of TMDC nanosheets, increasing, for example, the area of the sensing layer and consequently the amount of the receptor immobilized, thereby enhancing the sensing performance, with respect to single MoS_2_.

**Table 1:** 2D Mo-dichalcogenides sensors.

Sensor	Target	Sensor Platform	Technique	Linear Range/Sensitivity	References
AuNPs@MoS_2_	AA, DA, UA	GCE	DPV	50 μM–100 mM, 0.05–30 μM, 50 μM–40 mM	[76]
AuNPs/MoS_2_/GN	NO^-^_2_	GCE	CV, Amp	5.0 μM to 5.0 mM	[97]
2D/MoS_2_	Tyrosine	AuBSPE	CV	1580 μA·mM^−1^·cm^−2^	[49]
MoS_2_ NSs/N-GN	DA	GCE	CV	3.2 μM–5.68 mM/75.49 μA·mM^−1^·cm^−2^	[77]
ssDNA/MoS_2_-PANI	ssDNA	Pt	DPV	10^−15^–10^−6^ M	[90]
ssRNA/AuNPs@MoS_2_-Ti_3_C_2_	miRNA-182	GCE	DPV	1 fM–0.1 nM	[102]
Au-Pd/MoS_2_	H_2_O_2_	GCE	DPV, Amp	0.8 µM–10 mM/−0.5–20 mM	[60]
AgCl/MoS_2_	chloramphen	ITO	CV, Amp	4–531 µM/3802 µA mM^−1^ cm^−2^	[106]
MoS_2_	UA	Flexible	DPV, Amp	10–400 μM/98.3 ± 1 nA μM^−1^	[93]
2D-MoS_2_	Cortisol	EIS		1–500 ng/mL	[75]
MoS_2_	H_2_O_2_	CC	CV, Amp	5–3000 μM/5.3 μA mM^−1^ cm^−2^	[61]
SrMoSe_2_	MTZ	GCE	DPV	0.05–914.92 μM/1.13 μA μM^−1^ cm^−2^	[105]
MoSe_2_–GN	PDGF-BB	GCE	DPV	0.0001–1 nM	[96]
HEG-MoSe_2_	NADH	GCE	Amp	1–280 μM/0.0814 µA⋅µM^−1^⋅cm^−2^	[100]
MoSe_2_-GN/Ni	DA	-	CV	0.01–10 μM	[78]
S-MoSe_2_/NSG/Au/MIPs	DA	GCE	CV, DPV	0.05 μM–1000 μM	[83]
AuNPs/SiO_2_@MoSe_2_	DNA	GCE	CV, DPV	0.1 fM–100 pM	[91]
Mb@MnMoSe_2_	H_2_O_2_	GCE	CV, Amp	0.09–60 μM/222.78 A cm^−2^ mM^−1^	[63]
MoSe_2_-HGNs	SCCA	GCE	PhotoElectr	1 pg∙mL^−1^ −50 ng∙mL^−1^	[103]
Ag_2_Se-MoSe_2_-GSH	DA	GCE	Amp	0.05−1110 μM/-	[79]
AuNPs-MoSe_2_-GN	CEA	GCE	CV, DPV	+0.001−100 ng∙mL^−1^/-	[84]
MoS_2_-Thi-AuNPs	MicroRNA-21	GCE	SWV	1.0 pM to 10.0 nM/-	[115]
MoS_2_–Au@Pt	Glucose	GCE	CV	10 mM to 3 mM/-	[116]
Mn-MoS_2_	DA	PGS	EIS, DPV	-/-	[117]
FMNs/MoS_2_	PIK3CA Gene	GCE	CV, DPV	10^−16^ mol l^−1^ to 10^−8^ mol l^−1/^-	[118]

Recently, 2D layered molybdenum sulfide nanosheets have shown great potential through combination with DNA as a biorecognition medium, opening new opportunities to develop highly sensitive and specific electrochemical sensors. The use of specific short ssDNA sequences like aptamers has been proven to bind well with the unique transduction properties of 2D MoS_2_ nanosheets to realize aptasensing devices having a high response, without labeling or any use of amplifiers, even at very low concentrations (up to 10^−15^ M) of target DNA [90,119].

With respect to TDMCs, electrochemical sensors based on 2D-Mo oxides (see Table 2) are much less reported in the literature. This finding can be explained considering that it is complicate to synthesize Mo oxides nanosheets with very few layers, a prerequisite for obtaining high sensing performances.

**Table 2:** 2D Mo-oxide sensors.

Sensor	Target	Sensor Platform	Technique	Linear Range/Sensitivity	References
MoO_3_-GO	H_2_O_2_	GCE	-	0.92 μM–2.46 mM/391.32 μA mM^−1^ cm^−2^	[66]
MoO_3_	NO₂⁻	GCE	CV	-	[98]
α-MoO_3_	H_2_O_2_	-	CV, EIS, Amp	0.4 μM–57.6 mM/168.72 μA mM^−1^ cm^−2^	[64]
MoO_3_·2H_2_O-GN	Thiourea	GCE	CV	2.40 × 10^−3^–19.3 × 10^−3^ M	[120]

2D-MoO_3_ has, however, been reported for the electrochemical sensing of inorganic species (nitrite ions, [98]), hydrogen peroxide [64,66], and larger organic biomolecules (thiourea, [120]). To improve the weak conductivity, which restricts its application as an electrochemical sensor, the modification with graphene or graphene oxide has been accomplished, leading to nanocomposite-modified electrodes with a higher electron transfer rate than those with a bare electrode. The consequent performance enhancement has been further related to synergistic effects between layered carbon nanostructures and MoO_3_⋅2H_2_O, which increase the dispersion of the electrochemical active phase.

Among metal carbides, an emerging class of electrode materials employed for various electrochemical applications, 2D-MoC_2_ has been recently proposed for interesting applications in the field of electrochemical biomolecule sensing [86,92,99,101]. Table 3 focuses attention on these novel 2D Mo-carbide electrochemical sensors.

**Table 3:** 2D Mo-carbide sensors.

Sensor	Target	Sensor Platform	Technique	Linear Range/Sensitivity	References
MIP-Mo_2_C	FA	GCE	DPV	-	[86]
NCS/Mo_2_C	microRNA	GCE	CV, DPV, EIS,	1 fM–1 nM	[92]
Luminol-AuNPs@Mo_2_C	α-fetoprotein	GCE	ECL	0.1 pg·mL^−1^–30 ng·mL^−1^/-	[101]
MWCNTs-Mo_2_C	RIF	GCE	CV, DPV, CC	0.5–74 μM/-	[99]

However, most of the proposed sensors have been designed and fabricated at lab-scale, and are not suitable for industrial-scale production. It is obvious then that the main challenge for the future is the fabrication of reliable sensors for biomolecules that can be used in the field for real sample analysis. This involves the reproducible synthesis of 2D-Mo nanomaterials, as well as sensor miniaturization and their integration into labs on a chip. 

A further very interesting aspect to highlight from a future perspective is the improvement of the electrochemical characteristics of 2D-Mo nanomaterials through functionalization. As an example, Chu et al. introduced a method for functionalizing MoS_2_ using cysteine [121]. The introduced functional groups can contribute to improving the structural stability of 2D-Mo nanomaterials and their chemical/electrochemical reactivity, making them more promising for the desired electroanalytical applications. 

It appears then, that although realizing on-line real-time detection still has a long way to go, the development of more reliable 2D-Mo nanomaterial synthesis and chemical modification will be the routes leading to electrochemical sensor devices with high sensitivity, long-term stability, selectivity, and reliability, opening new ways for the highly effective detection of biomolecules for clinical diagnosis.

## 7. Conclusions

In summary, the preparation, morphological, and structural properties of Mo-based layered nanostructures have been reviewed. Due to their peculiar atom arrangement with anisotropic basal, and edge, planes, crystal structure, size, and defects, coupled with the chemical characteristics of the Mo center (e.g., multiple oxidation states), these two-dimensional nanostructures present good electrochemical properties. Their features have been exploited in potential applications for electrochemical biomolecule detection in physiological solution (blood, urine, sweat, saliva) fluids.

Apart from the good sensing capability above demonstrated, advantages in using these materials as sensor electrodes come from their simple synthesis and low-cost. On the other hand, stability and reproducibility of the synthesized products are still to be confirmed and improved. In addition, large scale production is questionable and should be verified. 

More research activity is necessary before these nanomaterials can be competitive with more conventional materials. Nevertheless, the progress made in this research area could be beneficial to overcoming the above cited drawbacks. This could make these sensors more competitive in the market in the near future.
